# Enhanced Radiological Detection of a Corticotroph Adenoma Following Treatment With Osilodrostat

**DOI:** 10.1210/jcemcr/luaf200

**Published:** 2025-10-07

**Authors:** Zin Htut, Lisa Yang, James MacFarlane, Daniel Gillett, Mark Gurnell, Florian Wernig

**Affiliations:** Division of Diabetes, Endocrinology and Metabolism, Imperial College London, London, UK; Department of Endocrinology, Imperial College Healthcare NHS Trust, London, UK; Department of Endocrinology, Cambridge University Hospitals NHS Foundation Trust, Cambridge, UK; Department of Endocrinology, Cambridge University Hospitals NHS Foundation Trust, Cambridge, UK; Cambridge Endocrine Molecular Imaging Group, University of Cambridge and NIHR Cambridge Biomedical Research Centre, Cambridge University Hospitals NHS Foundation Trust, Cambridge, UK; Cambridge Endocrine Molecular Imaging Group, University of Cambridge and NIHR Cambridge Biomedical Research Centre, Cambridge University Hospitals NHS Foundation Trust, Cambridge, UK; Department of Nuclear Medicine, Cambridge University Hospitals NHS Foundation Trust, Cambridge, UK; Department of Endocrinology, Cambridge University Hospitals NHS Foundation Trust, Cambridge, UK; Cambridge Endocrine Molecular Imaging Group, University of Cambridge and NIHR Cambridge Biomedical Research Centre, Cambridge University Hospitals NHS Foundation Trust, Cambridge, UK; Division of Diabetes, Endocrinology and Metabolism, Imperial College London, London, UK; Department of Endocrinology, Imperial College Healthcare NHS Trust, London, UK

**Keywords:** Cushing disease, osilodrostat, [11C]methionine PET, corticotroph adenoma

## Abstract

In approximately 30% of patients with Cushing disease, pituitary magnetic resonance imaging (MRI) does not reliably identify a corticotroph adenoma. Importantly, surgical remission rates are >2.5 fold higher for microadenomas that are radiologically visible on preoperative imaging when compared with “MRI-negative” cases. We describe a 42-year-old woman with Cushing disease, in whom MRI findings at presentation were equivocal with no clear adenoma visualized. She was initially treated with metyrapone, which resulted in partial biochemical control of hypercortisolism. After switching to osilodrostat, there was a marked improvement in her symptoms and rapid normalization of cortisol levels. Following 3 months of eucortisolemia, [11C]methionine positron emission tomography (MET-PET) coregistered with volumetric MRI (MET-PET/MR^CR^) localized the site of the corticotroph tumor and the patient underwent successful transsphenoidal resection. She remains in full clinical and biochemical remission at >2 years postsurgery. This case suggests that a period of eucortisolemia induced by osilodrostat may facilitate localization of corticotroph microadenomas using functional (PET) imaging.

## Introduction

Cushing disease, caused by an ACTH-secreting pituitary adenoma, accounts for approximately 80% of endogenous Cushing syndrome [1]. Although transsphenoidal surgery remains the preferred treatment for the majority of patients, even in expert centers recurrence rates as high as 27% have been reported [2, 3]. Surgery is preferred over medical therapy because it offers the potential for definitive cure by directly removing the pituitary adenoma. In contrast, medical therapy is typically reserved for patients in whom surgery is contraindicated, incomplete, or has failed to achieve remission. Linked to this, magnetic resonance imaging (MRI) fails to detect an adenoma in approximately one third of cases [4]. In a recent systematic review, postsurgical remission rates were 2.63-fold higher (95% CI, 2.06-3.35) for MRI-detected corticotroph adenomas when compared with “MRI-negative” cases [5]. Several alternative magnetic resonance sequences have therefore been proposed to aid tumor localization (including dynamic and volumetric [eg, gradient recalled echo MRI]), but these still fail to detect a significant proportion of microcorticotropinomas [6, 7]. Accordingly, molecular (functional) imaging with positron emission tomography (PET) radiotracers that target key properties of corticotroph adenomas (eg, [11C]methionine [MET-PET], [18F]fluoroethyltyrosine, or [68Ga]DOTA-corticotropin-releasing hormone PET) has been proposed as an additional tool for localizing corticotroph tumors that evade detection on conventional MRI [6-10].

Medical therapy is often required for patients in whom surgery is not an immediate option or when there is persistent hypercortisolism postoperatively [11]. Cortisol-lowering treatment may also be considered before surgery to reduce morbidity and perioperative complications [11]. An important recent addition to the armory of medications used to treat Cushing syndrome is osilodrostat, a potent oral inhibitor of the key adrenal steroidogenic enzyme 11β-hydroxylase [12, 13].

Here, we describe how preoperative medical therapy with osilodrostat yielded dual benefits in a patient with inconclusive primary imaging: (1) rapid and effective control of hypercortisolism and (2) facilitation of the localization of a previously occult microcorticotroph adenoma using MET-PET coregistered with volumetric MRI (MET-PET/MR^CR^).

## Case Presentation

A 42-year-old woman presented with a 7-year history of progressive central weight gain, facial plethora, acne, worsening hypertension, depression, and proximal myopathy. Her symptoms had become more pronounced during the COVID-19 pandemic, leading to profound emotional distress and functional decline. She described feeling persistently tearful and fatigued, with markedly reduced energy levels that rendered her unable to work or care for her young child, and severely affecting her quality of life. She had no significant medical history and was taking amlodipine and the progesterone-only pill. On examination, her body mass index was 29.6 kg/m² and blood pressure was markedly elevated at 197/111 mm Hg. Clinical features consistent with hypercortisolism included easy bruising, centripetal adiposity, and proximal muscle wasting. Initial laboratory evaluation was unremarkable; however, her hemoglobin A1c was at the upper end of normal (41 mmol/mol or 5.9%).

## Diagnostic Assessment

**Figure 1.**
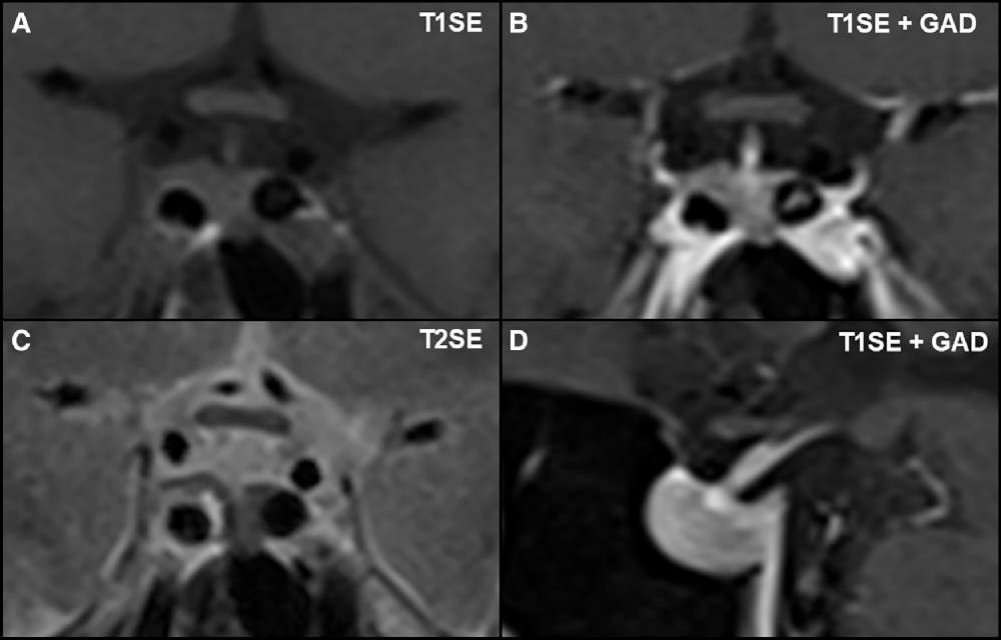
Pituitary MRI at initial presentation. No discrete adenoma is visible on T1-weighted coronal precontrast (A) and postcontrast (B), T2-weighted coronal (C), and T1-weighted sagittal postcontrast (D) sequences. The sellar anatomy appears asymmetric, consistent with a medially positioned left internal carotid artery.

Biochemical testing confirmed ACTH-dependent Cushing syndrome (Table 1). Cortisol levels following overnight and 48-hour dexamethasone suppression were elevated at 8 µg/dL (SI: 219 nmol/L) and 16 µg/dL (SI: 434 nmol/L), respectively (reference range: < 1.8 µg/dL [SI: < 50 nmol/L]). Plasma ACTH concentrations ranged from 36 to 55 ng/L (SI: 7.9-12.1 pmol/L) (reference range: 10-30 ng/L [SI: 2.2-6.6 pmol/L]), consistent with an ACTH-driven process. Urinary free cortisol (UFC) was markedly elevated at 690.95 µg/24 hours (SI: 1907 nmol/24 hours) (reference range: 18-98 µg/24 hours [SI: 50-270 nmol/24 hours]). Late-night salivary cortisol and cortisone levels were also elevated at 0.95 µg/dL (SI: 26.2 nmol/L) (reference range: < 0.09 µg/dL [SI: < 2.6 nmol/L]) and 2.7 µg/dL (SI: 74.5 nmol/L) (reference range: < 0.7 µg/dL [SI: < 18 nmol/L]) respectively. Inferior petrosal sinus sampling excluded an ectopic source of ACTH production (central-to-peripheral ACTH ratio: baseline 18.60, 0 minutes 18.4, peak at 2 minutes 94.9, 5 minutes 42.4, 10 minutes 22.3) (Table 2). However, pituitary MRI findings were inconclusive, with no definite adenoma identified. In addition, the left intracavernous carotid artery encroached medially, creating a narrow intercarotid window with distortion of normal pituitary anatomy (Fig. 1). Given these findings, the decision was made to initiate cortisol-lowering therapy and to reassess imaging appearances after a period of biochemical normalization.

**Table 1.** Biochemical investigations at diagnosis confirming ACTH-dependent Cushing syndrome

Tests	Results	Reference Range
Overnight dexamethasone suppression test (ONDST)	Cortisol: 8 µg/dL (SI: 219 nmol/L)	<1.8 µg/dL (SI: < 50 nmol/L)
48-hour dexamethasone suppression test (DST)	Cortisol: 16 µg/dL (SI: 434 nmol/L)	<1.8 µg/dL (SI: < 50 nmol/L)
ACTH	36-55 ng/L (SI: 7.9-12.1 pmol/L)	10-30 ng/L (SI: 2.2-6.6 pmol/L)
24-hour urinary free cortisol (UFC)	690.95 μg/24 h (SI: 1907 nmol/24 h)	18-98 µg/24 h (SI: 50-270 nmol/24 hours)
Late-night salivary cortisol late-night salivary cortisone	0.95 µg/dL (SI: 26.2 nmol/L) 2.7 µg/dL (SI: 74.5 nmol/L)	<0.09 µg/dL (SI: <2.6 nmol/L) <0.7 µg/dL (SI: <18 nmol/L)

Results are reported in both conventional and SI units with reference ranges shown in parentheses.

**Table 2.** Results of inferior petrosal sinus sampling (IPSS)

Time	Plasma ACTH		
(min)	Left petrosal sinus	Right petrosal sinus	Peripheral vein
−5	1159 ng/L (255 pmol/L)	144 ng/L (32 pmol/L)	62.3 ng/L (14 pmol/L)
0	1147 ng/L (253 pmol/L)	222 ng/L (49 pmol/L)	62.3 ng/L (14 pmol/L)
2	5257 ng/L (1157 pmol/L)	2159 ng/L (475 pmol/L)	55.4 ng/L (12.2 pmol/L)
5	3677 ng/L (810 pmol/L)	2976 ng/L (655 pmol/L)	86.8 ng/L (19 pmol/L)
10	2251 ng/L (496 pmol/L)	545 ng/L (120 pmol/L)	101 ng/L (22 pmol/L)

Time	Plasma cortisol		
(min)	Left petrosal sinus	Right petrosal sinus	Peripheral vein
−5	24.94 μg/dL (668 nmol/L)	25.30 μg/dL (698 nmol/L)	23.56 μg/dL (650 nmol/L)
0	25.08 μg/dL (692 nmol/L)	24.07 μg/dL (664 nmol/L)	23.34 μg/dL (644 nmol/L)
2	23.31 μg/dL (643 nmol/L)	24.32 μg/dL (671 nmol/L)	23.78 μg/dL (656 nmol/L)
5	21.97 μg/dL (606 nmol/L)	23.67 μg/dL (653 nmol/L)	23.23 μg/dL (641 nmol/L)
10	27.62 μg/dL (762 nmol/L)	26.17 μg/dL (722 nmol/L)	25.26 μg/dL (697 nmol/L)

Time	Plasma prolactin		
(min)	Left petrosal sinus	Right petrosal sinus	Peripheral vein
−5	1835 mU/L (86 μg/L)	356 mU/L (17 μg/L)	251 mU/L (11 μg/L)
0	1725 mU/L (81 μg/L)	498 mU/L (23 μg/L)	248 mU/L (12 μg/L)
2	2151 mU/L (101 μg/L)	409 mU/L (19 μg/L)	240 mU/L (11 μg/L)
5	2239 mU/L (105 μg/L)	711 mU/L (33 μg/L)	246 mU/L (12 μg/L)
10	1883 mU/L (89 μg/L)	410 mU/L (19 μg/L)	244 mU/L (11 μg/L)

Central-to-peripheral ACTH gradients before and after corticotropin-releasing hormone (CRH) stimulation support a pituitary source of ACTH secretion. Reference cutoffs: basal ACTH gradient ≥2 and/or CRH-stimulated ACTH gradient ≥3 indicate central ACTH secretion.

## Treatment

**Figure 2.**
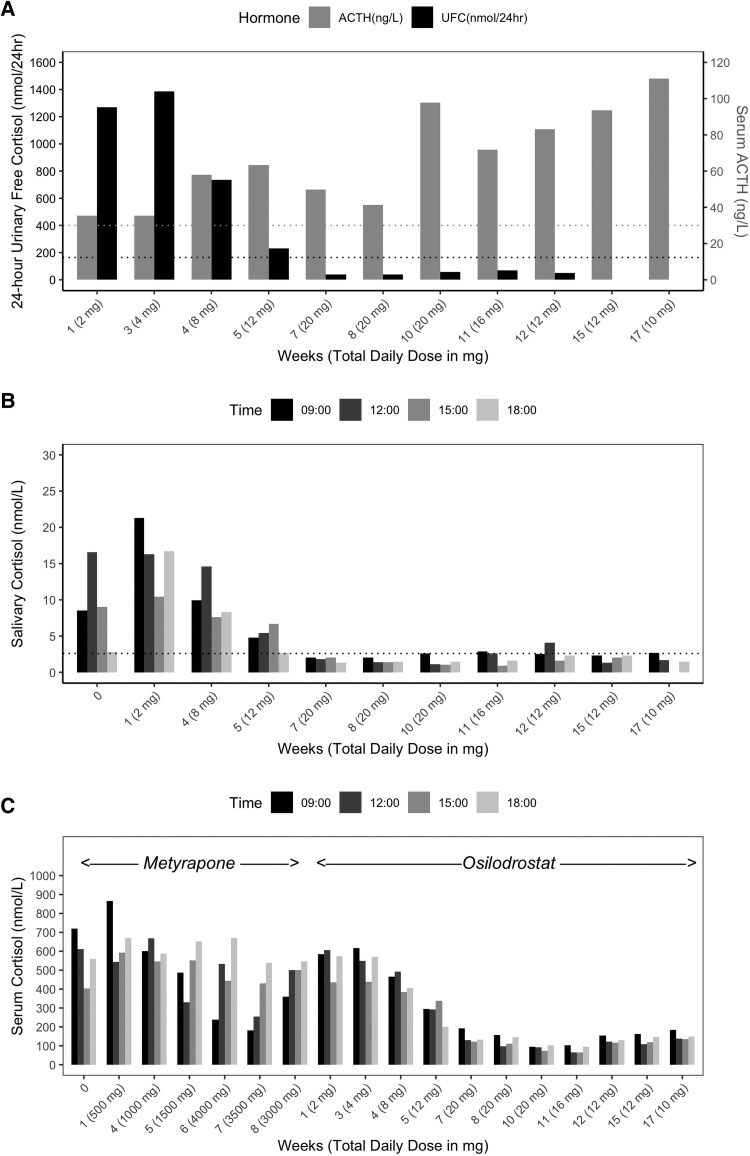
Bar charts illustrating changes in urinary, salivary, and serum cortisol, as well as serum ACTH, during medical treatment. (A) A 24-hour UFC (black bars, left y-axis) normalized during osilodrostat treatment, whereas serum ACTH (gray bars, right y-axis) increased. Dotted lines represent the upper limit of normal: 59.4 µg/24 hours (SI: 164 nmol/24 hours) for UFC and 30 ng/L (SI: 6.6 pmol/L) for ACTH. X-axis labels indicate treatment week and total daily osilodrostat dose. (B) Salivary free cortisol levels, collected alongside serum cortisol during a cortisol day curve (at 09:00, 12:00, 15:00, and 18:00), fully normalized with osilodrostat therapy. Bar shading from black to light gray denotes sampling time. The dotted line indicates upper limit of normal: 9.4 ng/dL (SI: 2.6 nmol/L). (C) Serum free cortisol levels during day curves showed inadequate control on escalating doses of metyrapone, with normalization achieved following initiation of osilodrostat.

The patient was started on metyrapone, but despite dose escalation up to 4000 mg daily, which was associated with significant nausea and malaise, she did not achieve eucortisolemia (Fig. 2C). She was therefore transitioned to osilodrostat, which rapidly normalized cortisol levels within 5 weeks at a maintenance dose of 6 mg twice daily (Fig. 2B and 2C). In contrast to metyrapone, osilodrostat was well-tolerated with no reported side effects. Serum cortisol and clinical status were closely monitored throughout, with no biochemical or clinical evidence of adrenal insufficiency.

**Figure 3.**
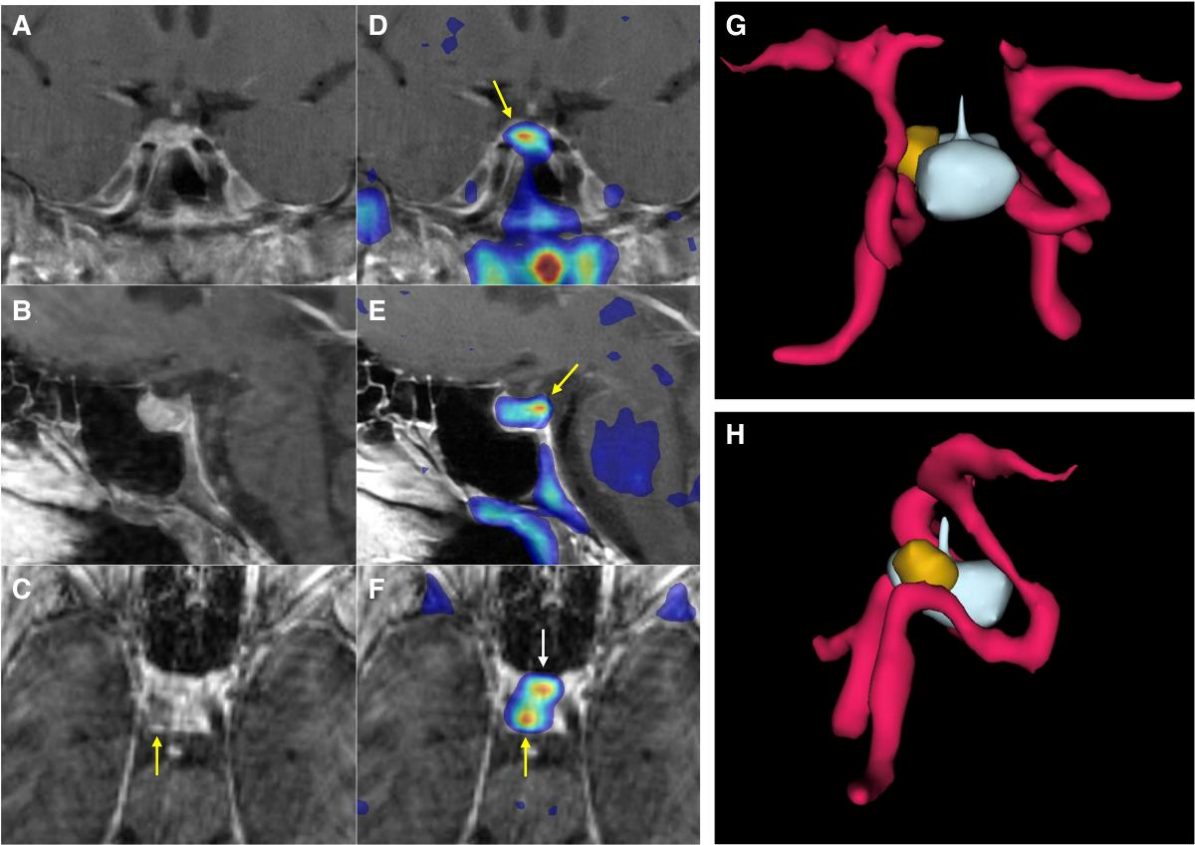
11C-Methionine PET/CT coregistered with volumetric MRI (MET-PET/MR^CR^) following treatment with osilodrostat. A subtle area of reduced gadolinium enhancement can now be appreciated on the right posterosuperior aspect of the gland (A-C). MET-PET/MR^CR^ confirms focal tracer uptake at this site (yellow arrows) and also within normal gland anteriorly (white arrow) (D-F). Three-dimensional reconstruction using CT, MRI, and PET datasets demonstrating the location of the corticotroph microadenoma which was confirmed at subsequent surgery (G-H).

ACTH levels progressively increased as the dose of osilodrostat was escalated (Fig. 2A). After 3 months of biochemical eucortisolism, she underwent Met-PET/MR^CR^, which revealed a distinct methionine-avid lesion in the right posterolateral aspect of the sella (Fig. 3). Imaging was performed as previously reported [7, 8, 14]. Conventional MRI findings remained stable, with no new abnormalities. As she remained clinically and biochemically eucortisolemic on osilodrostat, glucocorticoid supplementation was not required pre- or perioperatively.

## Outcome and Follow-up

At transsphenoidal surgery, abnormal tissue was resected from the site identified on MET-PET/MR^CR^. Histological examination revealed normal anterior pituitary tissue (adenohypophysis) with no evidence of a pituitary adenoma. Occasional cells showed possible Crooke's hyaline change. The Ki-67 proliferation index was very low (<1%). Despite the absence of histological confirmation of a corticotroph adenoma, the patient entered complete biochemical and clinical remission. Early postoperative cortisol was 3 µg/dL (SI: 82.8 nmol/L), prompting initiation of glucocorticoid replacement with prednisolone. Prednisolone was chosen for its longer half-life, enabling convenient once-daily dosing. We routinely monitor prednisolone levels to guide adjustment of replacement dosing. Prednisolone was successfully tapered over a period of 6 months, with biochemical confirmation of adrenal recovery. At 2 years postsurgery, the patient had no clinical features of hypercortisolism with sustained weight loss of >20 kg. Morning 09:00 cortisol and ACTH were consistent with ongoing eucortisolism. Serial late-night salivary cortisol and cortisone levels were normal, and cortisol was undetectable following a 1-mg overnight dexamethasone suppression test, confirming durable remission of Cushing disease.

## Discussion

Early transsphenoidal surgery remains the treatment of choice for most patients with Cushing disease, with the highest chance of cure achieved following a successful first operation [11]. However, even in expert centers, persistent or recurrent disease is diagnosed during follow-up, and is more likely when initial MRI has failed to identify a clear surgical target [5]. Reoperation carries increased technical difficulty and a higher risk of iatrogenic hypopituitarism, underscoring the importance of accurate preoperative localization of corticotroph adenomas. Our case illustrates a potential novel added benefit of a trial of primary medical therapy in a patient with Cushing disease and equivocal or negative MRI findings at initial presentation. Specifically, we have shown how osilodrostat, a potent inhibitor of 11β-hydroxylase, can achieve rapid normalization of cortisol levels, consistent with the findings of the LINC (LCI699 [osilodrostat] in Cushing disease) series of studies [15-17], and at the same time help reveal the location of the occult microcorticotropinoma. An important consequence of achieving effective adrenal blockade in our patient was the more than threefold accompanying rise in plasma ACTH levels (Fig. 2). We hypothesized that such an increase in tumoral activity might facilitate its detection using molecular (functional) imaging. MET-PET has been shown in several studies to facilitate localization of de novo and recurrent corticotroph adenomas [8, 18, 19] in a significant proportion of patients with equivocal or negative MRI findings. We have now shown that such an approach could potentially be enhanced by pretreatment with the potent 11β-hydroxylase inhibitor osilodrostat.

We also considered whether the rise in ACTH during osilodrostat therapy reflected increased tumor activity alone or was associated with a change in tumor size. In our case, ACTH rose significantly, likely reflecting enhanced secretory activity, whereas repeat conventional MRI remained stable, with no new abnormalities or interval changes. In the LINC 4 study, tumor volume data were available for 35 patients at both baseline and week 48. Among these, 40.0% had a ≥20% increase, 28.6% had a ≥20% decrease, and 31.4% had <20% change in tumor volume. These outcomes were observed in both microadenomas and macroadenomas, with no clear correlation to treatment duration or osilodrostat dose [20]. This variability suggests that osilodrostat does not exert a consistent effect on tumor volume.

Interestingly, although histopathological analysis did not confirm a corticotroph adenoma, this is a well-recognized finding and has been reported in a significant proportion of patients undergoing surgery for Cushing disease [21, 22]. Nonetheless, we consider the diagnosis of pituitary-dependent Cushing syndrome was clearly established by the clinical features, results of initial laboratory testing and findings at inferior petrosal sinus sampling (which demonstrated a clear central-to-peripheral ACTH gradient). In addition, abnormal tissue was identified intraoperatively at the site visualized on MET-PET and fully resected, and no other abnormal foci of tissue were seen. The patient has subsequently achieved complete and sustained clinical and biochemical remission, consistent with successful removal of an ACTH-secreting adenoma.

Recent case reports have raised concerns about prolonged adrenal insufficiency following extended osilodrostat use—an unexpected finding given the drug's short half-life [23-25]. Although adrenal insufficiency requiring temporary glucocorticoid replacement had been reported in clinical trials (most commonly in patients undergoing rapid dose escalation [12, 15, 16]), prolonged hypothalamopituitary-adrenal axis suppression resulting from supraphysiologic glucocorticoid replacement could also be contributory. For now, the exact mechanism of this observed phenomenon remains unclear. Our patient managed to wean glucocorticoid replacement postoperatively and did not demonstrate prolonged adrenal suppression; at the same time, clinical and biochemical testing confirmed full remission from Cushing disease.

This case supports the hypothesis that preoperative cortisol suppression may enhance the diagnostic accuracy of molecular (functional) imaging in Cushing disease, particularly in cases with inconclusive MRI findings. If validated in prospective studies, this approach could refine surgical planning and potentially lead to better surgical success and durable clinical outcomes.

## Learning Points

- Approximately 30% of corticotroph adenomas causing Cushing disease are not readily localized on conventional pituitary MRI.
- Functional imaging modalities such as MET-PET/MRCR can improve detection of previously occult pituitary adenomas in Cushing disease.
- A period of medical pretreatment with osilodrostat, with consequent reduction in negative feedback by glucocorticoid at the hypothalamic-pituitary level, may augment tumor localization by molecular imaging.

## Data Availability

Original data generated and analyzed during this study are included in this published article.
